# Potent Ameliorative Effects of Rosmarinic Acid on Tramadol-Induced Neurotoxicity in the Brain and Hippocampus; by Suppressing Oxidative stress, Apoptosis, ER stress, and Regulating Cognitive Functions

**DOI:** 10.1007/s12035-025-04892-8

**Published:** 2025-04-07

**Authors:** Onur Karaca, Hasan Şimşek, Nurhan Akaras, Cihan Gür, Mustafa İleritürk, Özge Kandemir, Sefa Küçükler, Şeyda Öte Karaca, Fatih Mehmet Kandemir

**Affiliations:** 1 Department of Anesthesiology and Reanimation, Faculty of Medicine, Aksaray University, Aksaray, Turkey; 2 Department of Physiology, Faculty of Medicine, Aksaray University, Aksaray, Turkey; 3 Department of Histology and Embryology, Faculty of Medicine, Aksaray University, Aksaray, Turkey; 4 Department of Medical Laboratory Techniques, Vocational School of Health Services, Atatürk University, Erzurum, Turkey; 5 Department of Animal Science, Horasan Vocational College, Atatürk University, Erzurum, Turkey; 6 Department of Food Processing, Aksaray Technical Sciences Vocational School, Aksaray University, Aksaray, Turkey; 7 Department of Veterinary Biochemistry, Faculty of Veterinary, Atatürk University, Erzurum, Turkey; 8 Department of Physical Medicine and Rehabilitation, Aksaray Training and Research Hospital, Aksaray, Turkey; 9 Department of Medical Biochemistry, Faculty of Medicine, Aksaray University, Aksaray, Turkey

**Keywords:** Apoptosis, Cognitive function, Neurotoxicity, Oxidative stress, Rosmarinic acid, Tramadol

## Abstract

Tramadol (TRM) is a synthetic opioid analgesic that acts on the central nervous system and is used to treat moderate or severe pain. However, the incidence of its abuse is increasing. Rosmarinic acid (RA) is a natural flavonoid known for its antioxidant, anti-inflammatory, and neuroprotective properties. In this study, we determined the ameliorative effects of RA against TRM-induced neurotoxicity. Thirty​​​​​​​ five rats were divided into 5 groups; control, RA, TRM, TRM + RA25 and TRM + RA50. TRM 50 mg/kg was administered intraperitoneally, and RA 25 and 50 mg/kg doses were administered by oral gavage for 14 days. Water Maze Test (WMT) was performed to assess cognitive function. Oxidative stress, inflammation, endoplasmic reticulum (ER) stress, apoptosis damage pathways, glial fibrillary acidic protein (GFAP), and brain-derived neurotrophic factor (BDNF) activities were determined in brain and hippocampus tissues. The structural and functional integrity of the tissues were also analyzed. RA decreased TRM-induced increased ​​​​​​​oxidative stress, inflammation, ER stress, and apoptotic damage levels. In addition, it improved neuronal survival and activity by bringing BDNF and GFAP activities closer to normal in brain tissue. RA restored the structural properties of brain and hippocampus tissues disrupted by tramadol. These findings were also demonstrated using WMT, which improved the arrival time to the quadrant in which the platform was located and the time spent in the quadrant. RA reduces TRM-induced neurotoxicity by reducing inflammation, oxidative stress, ER stress, and apoptotic damage and increases neuronal survival and activity.

## Introduction

Tramadol (TRM) is a synthetic analgesic drug approved in 1995 by the Food and Drug Administration (FDA), exhibiting both opioid and non-opioid properties and acting mainly through inhibition of the central nervous system [1]. TRM, a drug commonly used to reduce trauma, alleviate chronic pain, and improve quality of life, is included in many medical guidelines. However, the pharmacologic effects of this drug remain controversial. TRM belongs to a group of multi-receptor drugs that functions as a µ1 opioid receptor agonist, monoamine reuptake inhibitor, and inhibitor of ligand-gated ion channels and some specific protein-coupled receptors [2].

Substance addiction, which causes serious physical and mental health problems, has become an important public health problem. Among the sources of addiction, opioids, such as morphine, which account for a large share of narcotics, draw attention [3]. The International Narcotics Control Board's 2013 review report noted that TRM use has become problematic in almost half of the countries reviewed (32 out of 77 countries). It also reported that TRM use is increasingly being misused and is beginning to manifest itself in the form of intentional overdose or poisoning [4]. Among the factors contributing to its increasing abuse is that it is also effective in increasing male sexual activity [5]. Another cause of concern is seizures due to TRM overdose, which accounts for 8% of all drug-related seizures. Seizures have been described in several reports after chronic use of TRM for pain management, recreational use, or after a single administration of > 500 mg [6]. The most prominent side effects related to TRM toxicity are neurotoxicity, cardiotoxicity,​​​​​​​ and gastrointestinal symptoms. In TRM-induced neurotoxicity, symptoms begin to appear within 24 h, and usually, these symptoms are generalized tonic–clonic seizures [7]. Long-term opioid use can lead to structural changes and apoptosis in neurons. Chronic opioid use increases reactive oxygen species (ROS) and oxidative stress. Moreover, ROS production can trigger inflammatory processes, and exposure to opioid receptor agonists is associated with the activation of apoptotic mechanisms [8]. With TRM administration, increased ROS production can lead to cell damage. It has also been associated with the activation of apoptotic mechanisms [9]. Such​​​​​​​ side effects of synthetic drugs, such as TRM, have become a significant burden for healthcare systems and a cause for concern. Therefore, medicinal plants are a powerful alternative option for reducing side effects because they are natural and have virtually no side effects [10].

Recent studies have shown an increasing interest in pharmacologically active compounds such​​​​​​​ as alkaloids, glycosides, flavonoids, phenolic compounds, and cannabinoids, in natural plants. Flavonoids have attracted considerable attention for their anti-inflammatory, antiviral, antibacterial, and antioxidant properties [11]. Rosmarinic acid (RA) is a polyphenolic secondary metabolite found in many plant species. This compound has several pharmacological properties, such as prevention of low-density lipoprotein (LDL) oxidation, inhibition of cell proliferation, and antiallergic effects. The drug also has broad therapeutic potential due to its anti-inflammatory, antioxidant, astringent, antimutagenic, antibacterial, and antiviral effects [12]. However, RA is a powerful agent with direct free radical scavenging ability. It protects neurons by cooperating with sensitive signaling pathways and neurotransmitter work [13]. RA protects astrocytes and macrophages against apoptosis and ROS-induced damage by increasing brain antioxidants and decreasing cytokine and inflammatory mediators [14].

In the literature, there are no available reports or data on the effects of RA against TRM-induced neurotoxicity​​​​​​​, and therefore, this study aimed to examine the ameliorative effects of RA against TRM-induced neurotoxicity in male rats.

## Material – Methods

### Chemicals

TRM (Contramal® Ampul 100 mg, Abdi İbrahim, Istanbul) and RA (Sigma, Cas No: 20283–92-5, purity 96%) were purchased from commercial companies. All other chemicals were obtained from Sigma.

### Ethical Approval

Ethical approval was obtained from Necmettin Erbakan University Local Animal Experiments Ethics Committee (08.02.2024, 2024–06). All experimental procedures were carried out by the European Directive 2010/63/EU for animal experiments. All procedures performed in this study involving animals complied with the ARRIVE guidelines.

### Groups and Experimental Procedures

Thirty-five male *Wistar albino* rats (220–250 g, 8 weeks old) were obtained from Necmettin Erbakan University KONÜDAM Experimental Medicine Application and Research Center (Konya/Turkey). Regarding the use of male rats, this choice was made to minimize hormonal variations that could influence the study outcomes, as fluctuations in female hormonal cycles may affect oxidative stress, apoptosis, and cognitive function. This approach aligns with the SAGER (Sex and Gender Equity in Research) guidelines, which emphasize the importance of clear reporting on sex-based decisions in study designs. Rats were housed under standard laboratory conditions. Rats were randomly divided into five groups (n = 7).

1. Control (C): For 14 days, saline was administered intraperitoneally once daily for 14 days.​​​​​​​
2. Tramadol (TRM): TRM 50 mg/kg was administered intraperitoneally once daily.
3. Rosmarinic Acid (RA): RA 50 mg/kg was administered once daily via oral gavage for 14 days.
4. Tramadol + Rosmarinic Acid-25 (TRM + RA25): TRM was administered at 50 mg/kg intraperitoneally once daily for 14 days. Half an hour after TRM administration, 25 mg/kg RA was administered orally once daily for 14 days.
5. Tramadol + Rosmarinic Acid-50 (TRM + R50): TRM was administered at 50 mg/kg intraperitoneally once daily for 14 days. Half an hour after TRM administration, RA (50 mg/kg) was administered orally once daily for 14 days.

The doses of TRM and RA [15, 16] used in this study and sample size were selected based on previously published literature that demonstrated their respective neurotoxic and neuroprotective effects in rodent models. These doses were chosen to replicate conditions that closely mimic clinical scenarios of TRM-induced toxicity while maintaining safe and effective levels for RA intervention.

### Behavioral Tests

The Morris Water Test (MWT) was used to measure spatial learning and memory abilities. This test consisted of a circular pool 45 cm deep and 150 cm wide. The pool is divided into four equal quadrants with a 10 × 10 cm hidden platform located in the center of the north quadrant. During the experiment, the test room was illuminated by a constant light source, and various figures and objects were hung on the walls to provide visual cues for the rats. All trials were recorded and analyzed by a video camera mounted on the ceiling. In the experiment, rats were subjected to acquisition and probe tests. In the acquisition test, rats were placed in the pool in different quadrants once a day for 3 days (15–17. days). For the first three days, the platform was placed 2 cm above the water level. On day 4 (18. day), the water height was adjusted so that it was 2 cm above the platform. Each trial lasted 60 s and rats were allowed to stay on the platform for 15 s once they found the platform. If they could not find the platform at the end of 60 s, they were placed on the platform for 15 s. The probe test (day 5) was performed 24 h after the last acquisition trial (day 19). At this stage, the platform was removed from the pool, and the water was dyed with a non-toxic food coloring. The rats were allowed to swim freely for 60 s. In the probe test, the time to first reach the quadrant where the platform was placed in the acquisition test but not in the probe test, the time spent in this quadrant, and the number of transitions to the quadrant were analyzed. The proposed method is modified from the method of Topuz et al. [17].

### Tissue Collection

24 h after the last procedure (day 20), rats were decapitated under mild sevoflurane (Sevorane®; Queenborough, UK) anesthesia, and brain (cerebral cortex) and hippocampus tissues were removed. One section of tissue was stored at −80 °C for biochemical analysis, and the other section was placed in 10% formaldehyde solution for histological analysis.

### Lipid Peroxidation Analysis

To determine the level of lipid peroxidation in brain and hippocampus tissues, the absorbance at 532 nm of the color produced by the reaction of malondialdehyde (MDA) and thiobarbituric acid was measured. For the analysis, tissues were homogenized in 1.15% potassium chloride (KCl) solution using a homogenizer. Homogenates were centrifuged at 4 °C and 1000xg for 15 min, and the supernatant was collected. The method developed by Placer et al.[18] was used to determine MDA levels.

### Antioxidants analysis

The antioxidant status in brain and hippocampus tissues was assessed by analyzing superoxide dismutase (SOD), catalase (CAT), glutathione peroxidase (GPx), and glutathione (GSH) levels. The supernatants used for the analysis of SOD, CAT, and GPx activities, as well as GSH levels, were obtained similarly as for lipid peroxidation. CAT activity was measured according to the method of Aebi [19], SOD activity according to the method of Sun et al. [20], GPx activity according to the method developed by Lawrence and Burk [21], GSH levels according to the method developed Sedlak and Lindsay [22]. The total protein content of the tissues required for the calculation of enzyme activities was determined by the method of Lowry et al. [23]

### qRT-PCR Analysis

Total RNA was isolated from brain and hippocampus tissues using QIAzol Lysis Reagent (79,306; Qiagen). Total RNA was then converted to cDNA using an RT2 First Strand Kit (330,404; Qiagen). All procedures were performed according to the manufacturer’s instructions. In the final step, the reaction was set up with RT2 SYBR® Green qPCR Mastermix (330,500; Qiagen), cDNAs, and primers for the genes of interest (Table 1) in Rotor-Gene Q (Qiagen) according to the manufacturer’s instructions. After the reaction was completed, genes were normalized to β-Actin using the 2^−ΔΔCT^ method [24].

**Table 1:** Primer sequences

Gene	Sequences (5’−3’)	Product Length	Accession No
NF-κB	F: AGTCCCGCCCCTTCTAAAAC R: CAATGGCCTCTGTGTAGCCC	106	NM_001276711.1
TLR-4	F: GCTCTGCCAAGTCTCAGATA R: GCTCTTCTAGACCCATGAAG	160	NM_019178.2
TNF-α	F: CTCGAGTGACAAGCCCGTAG R: ATCTGCTGGTACCACCAGTT	139	NM_012675.3
IL-1β	F: ATGGCAACTGTCCCTGAACT R: AGTGACACTGCCTTCCTGAA	197	NM_031512.2
nNOS	F: TGGAGACATCATTCTCGCAG R: GATGTGTAGTGAAGCCCTCA	140	NM_052799.2
RAGE	F: CTGAGGTAGGGCATGAGGATG R: TTCATCACCGGTTTCTGTGACC	113	NM_053336.2
NLRP3	F: TCCTGCAGAGCCTACAGTTG R: GGCTTGCAGCACTGAAGAAC	185	NM_001191642.1
eIF2-α	F: AGACCTGGATATGGTGCCTA R: CCTTCGTAACCATAGCAAGC	182	NM_019356.1
ATF-4	F: CTTCCTGAACAGCGAAGTGT R: ATAGCCAGCCATTCTGAGGA	171	NM_024403.2
ATF-6	F: TCAACTCAGCACGTTCCTGA R: GACCAGTGACAGGCTTCTCT	130	NM_001107196.1
PERK	F: GATGCCGAGAATCATGGGAA R: AGATTCGAGAAGGGACTCCA	198	NM_031599.2
IRE1	F: GCAGTTCCAGTACATTGCCATTG R: CAGGTCTCTGTGAACAATGTTGA	163	NM_001191926.1
Caspase-3	F: ACTGGAATGTCAGCTCGCAA R: GCAGTAGTCGCCTCTGAAGA	270	NM_012922.2
Bax	F: TTTCATCCAGGATCGAGCAG R: AATCATCCTCTGCAGCTCCA	154	NM_017059.2
Bcl-2	F: GACTTTGCAGAGATGTCCAG R: TCAGGTACTCAGTCATCCAC	214	NM_016993.2
BDNF	F: CATAACCCCGCACACTCTGT R: TCACCTGGTGGAACTTCCCG	90	NM_001270638.1
β-Actin	F: CAGCCTTCCTTCCTGGGTATG R: AGCTCAGTAACAGTCCGCCT	360	NM_031144.3

### Western-Blot Analysis

Western blotting was conducted on brain and hippocampal tissues, which were frozen post-extraction from rats. Then, the tissues were pulverized with liquid nitrogen and homogenized by adding RIPA buffer. Subsequently, homogenates were subjected to centrifugation at 16,000 g for 20 min. The total protein quantification was conducted with the Pierce BCA assay kit on the supernatant. Subsequently, proteins were diluted with 5X Laemmli sample buffer. SDS-PAGE was performed using a 10% gel concentration to separate proteins. Subsequently, separated proteins were transferred to PVDF membranes and incubated with 5% BSA in phosphate-buffered saline with 0.1% Tween 20 (PBS-T) for 1.5 h to reduce background interference. The membranes were washed five times with PBS-T and incubated at + 4 °C overnight with primary antibodies (1:500 dilution) at the end of this period. The membranes were washed five times with PBS-T and then treated for 1.5 h with an HRP-conjugated goat anti-mouse IgG secondary antibody (1:2000 dilution). The secondary antibody was removed and washed 5 times with PBS-T, and the bands were visualized with BioRad Clarity Max ECL substrate (Bio-Rad, Hercules, USA) and imaged with the Biorad Gel Doc XR + Imaging System (Bio-Rad, Hercules, USA). Densitometric analysis of the blots was performed using the ImageLab program (Bio-Rad, Hercules, USA). At least 3 replicate measurements were made from each sample.

#### Hematoxylin & Eosin

Brain and hippocampus tissue samples from rats were kept in 10% formalin solution for 48 ​​​​​​​hours for fixation. Tissue samples were embedded in paraffin blocks by passing through alcohol and xylol series using standard tissue tracking procedures. Subsequently, preparations were obtained by sectioning at a 5-micron thickness using a microtome. The preparations were stained with hematoxylin–eosin (HE), and the stained tissues were evaluated and photographed using a light microscope (Olympus Cx43; Japan). The results were evaluated as negative (-), mild ( +), moderate (+ +), and severe (+ +  + +) according to histopathologic changes under light microscope.

#### Immunohistochemical Analysis

3 µm thick sections taken from brain tissues were prepared for staining after being passed through xylene and alcohol series. Antigen retrieval was performed by keeping the sections in a citrate buffer. Then, it was kept in 3% hydrogen peroxide for 10 min. The sections, in which endogenous peroxidase activity was inhibited, were washed with phosphate-buffered saline (PBS) and then treated with protein block for 10 min. The primary antibody diluted with PBS was dropped onto the sections and kept in the refrigerator (+ 4 °C) overnight. Then, after washing with PBS three times for 5 min, they were treated with secondary antibody and Strepto Biotin, respectively. After each procedure, the DAB solution was dropped on the sections washed with PBS and waited until a brown color appeared. The sections were treated with Haris Hematoxylin for 5 min and washed again with PBS. The tissues were kept in 96% alcohol for 5 min, in absolute alcohol twice for 5 min, and in xylene twice for 5 min and then covered with entellan. Antibodies used for immunohistochemical staining: glial fibrillary acidic protein (GFAP) (Santa Cruz, Sc-33673) and cysteine–aspartic acid protease 3 (Casp-3) (Santa Cruz, Sc-56056). GFAP and Casp-3 immunopositivity were scored as follows: no change, ( −), mild change ( +), moderate change (+ +), and severe change (+ + +).

#### Statistical Analysis

The data obtained at the end of the study were analyzed using IBM SPSS software. Data are presented as mean ± standard deviation. Tukey’s post hoc tests and one-way analysis of variance were used for multiple comparisons of the data. Statistical significance was determined at p < 0.05, p < 0.01, p < 0.001 levels.

## Results

### Behavioral Tests Results

**Fig. 1:**
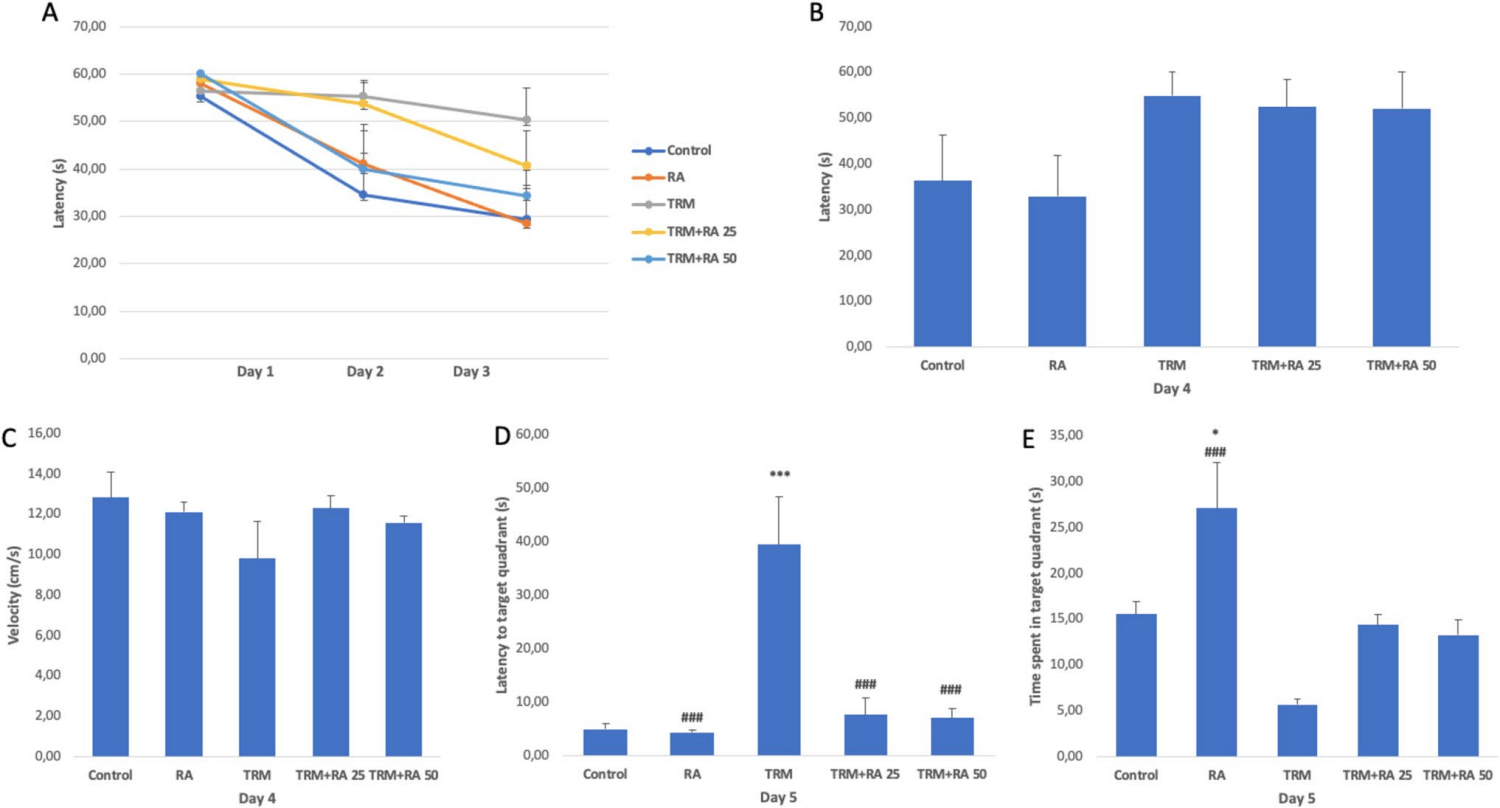
Effects of TRM and RA applications on cognitive functions. A: latency to platform during the first 3 days of the acquisition phase, B: latency to platform during the fourth day of the acquisition phase, C: Velocity on the fourth day of the acquisition phase, D: latency to the target quadrant in the probe phase, E: time spent in target quadrant in the probe phase. Statistical significance (Control vs others: *p < 0.05, **p < 0.01, ***p < 0.001, TRM vs others: #p < 0.05, ##p < 0.01, ###p < 0.001) was analyzed by One Way ANOVA

The MWT was used to assess learning memory in rats (Fig. 1). In the first three days of the acquisition phase, there was a gradual decrease in the groups except the TRM group, while this decrease was insignificant in the TRM group (Fig. 1A). On day 4, the time to reach the platform was higher in the TRM group than in the control group, but this difference did not reach significance. When RA was applied together with TRM, the applied times slightly detected between the groups when the velocities of the rats were compared (Fig. 1C). The time to reach the first quadrant on day 5, which is the probe phase, was longer in the TRM group than in the control group (p < 0.001). When RA was applied together with TRM, the occurrence of these events decreased (p < 0.001 at both doses) (Fig. 1D). Considering the time spent in the quadrant where the platform was located on day 5, which was also in the probe phase, less time was detected in the TRM group compared to the control group; however, this difference did not reach significance. Although there was an increase in this time in the groups administered RA compared with the TRM group, the difference did not reach significance (Fig. 1E).

### Oxidative Stress Results

**Fig. 2:**
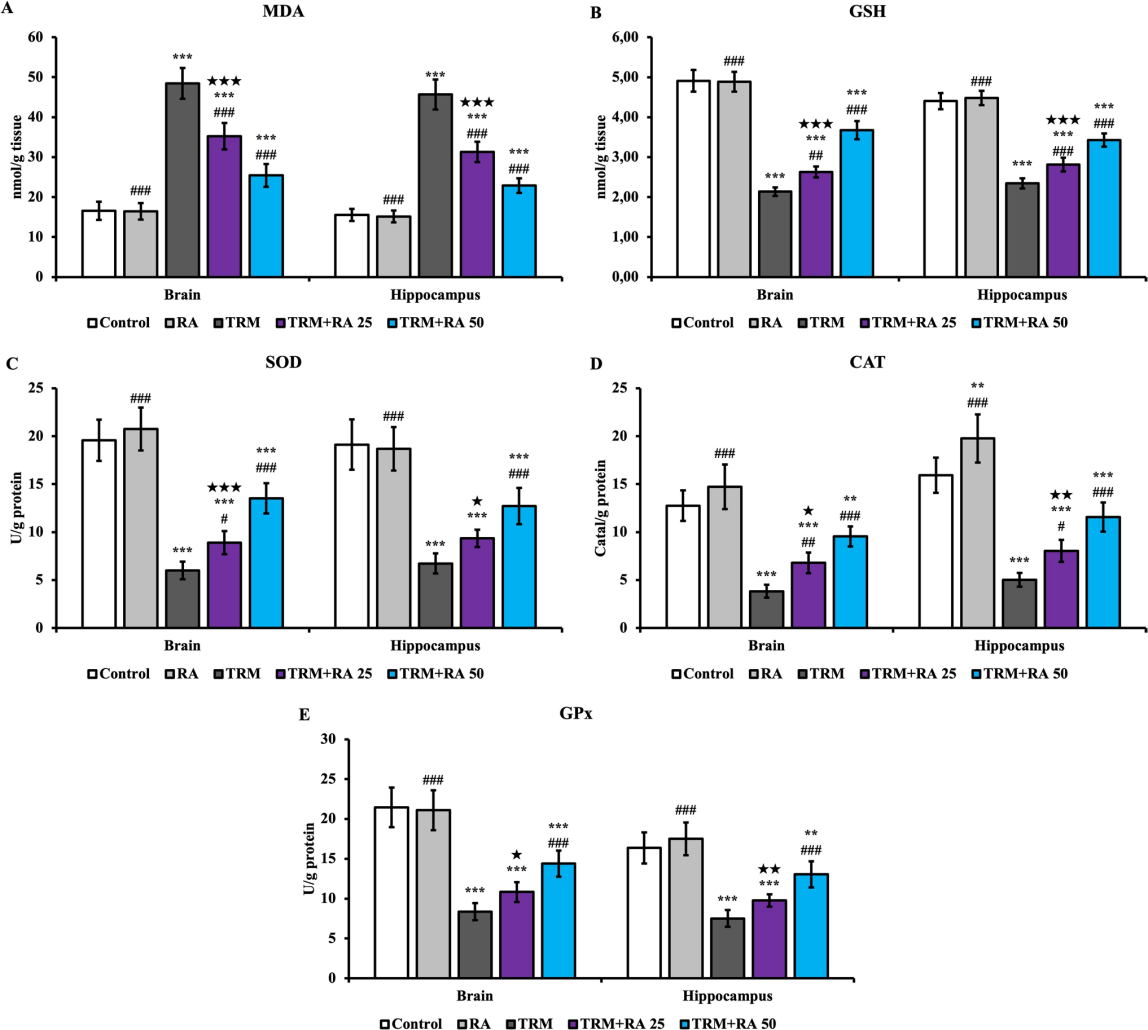
Effects of TRM and RA applications on MDA and GSH levels and SOD, CAT and GPx activities in the brain and hippocampus tissues of rats. Statistical significance (Control vs others: *p < 0.05, **p < 0.01, ***p < 0.001, TRM vs others: #p < 0.05, ##p < 0.01, ###p < 0.001, TRM + RA 25 vs TRM + RA 50: ★p < 0.05, ★★p < 0.01, ★★★p < 0.001) was analyzed by One Way ANOVA

The oxidant marker MDA levels (Fig. 2A) increased in both brain and hippocampus tissues owing to TRM (p < 0.001). When RA was administered together with TRM, MDA levels decreased compared with TRM (p < 0.001 at both doses), and RA was even more effective at higher doses (p < 0.001).

TRM administration decreased GSH (Fig. 2B) levels and SOD (Fig. 2C), CAT (Fig. 2D), and GPx (Fig. 2E) activities in both brain and hippocampus tissues (p < 0.001). RA administration in combination with TRM increased the levels and activities of these antioxidants in brain and hippocampus tissues. RA was even more effective at higher doses (significance details are given in Fig. 2).

### Endoplasmic Reticulum Stress Results

**Fig. 3:**
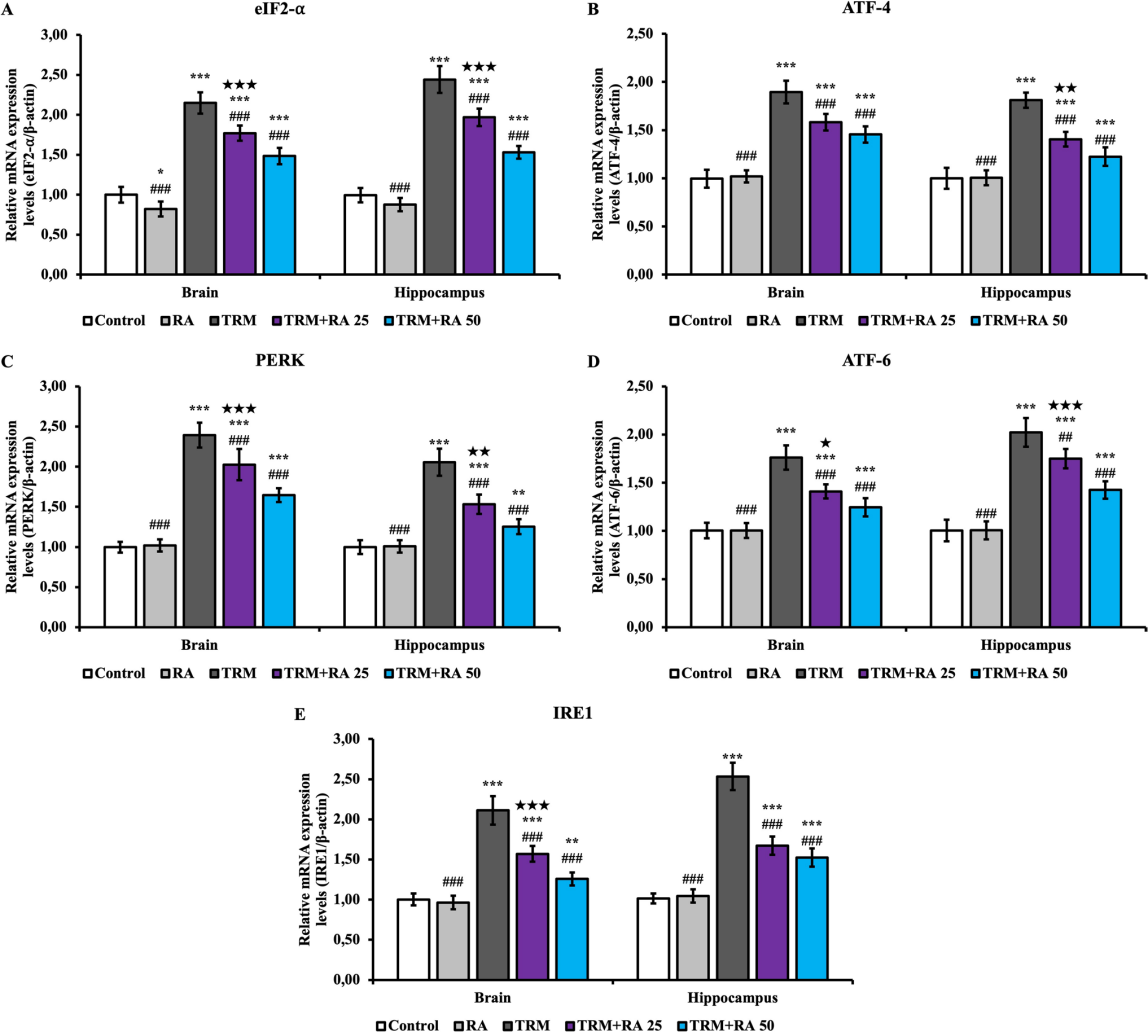
Effects of TRM and RA applications on eIF2**-⍺**, ATF-4, PERK, ATF-6 and IRE1 mRNA transcription levels in brain and hippocampus tissues of rats. Statistical significance (Control vs others: *p < 0.05, **p < 0.01, ***p < 0.001, TRM vs others: #p < 0.05, ##p < 0.01, ###p < 0.001, TRM + RA 25 vs TRM + RA 50: ★p < 0.05, ★★p < 0.01, ★★★p < 0.001) was analyzed by One Way ANOVA

To determine the level of endoplasmic reticulum (ER) stress in brain and hippocampus tissues, eukaryotic translation initiation factor 2 alpha subunit (eIF2-⍺) (Fig. 3A), activating transcription factor 4 (ATF-4) (Fig. 3B), protein kinase R (PKR)-like endoplasmic reticulum kinase (PERK) (Fig. 3C), activating transcription factor 6 (ATF-6) (Fig. 3D), and inositol-requiring enzyme 1 (IRE1) (Fig. 3E) mRNA transcription levels were determined. TRM treatment increased eIF2-⍺, ATF-4, PERK, ATF-6, and IRE1 mRNA transcription levels in brain and hippocampus tissues (p < 0.001). RA administration reversed the effect of TRM and decreased the levels (for ATF-6 in hippocampus tissue at 25 mg/kg dose; p < 0.01, for all other parameters in both tissues and at both doses; p < 0.001).

### Inflammation Results

**Fig. 4:**
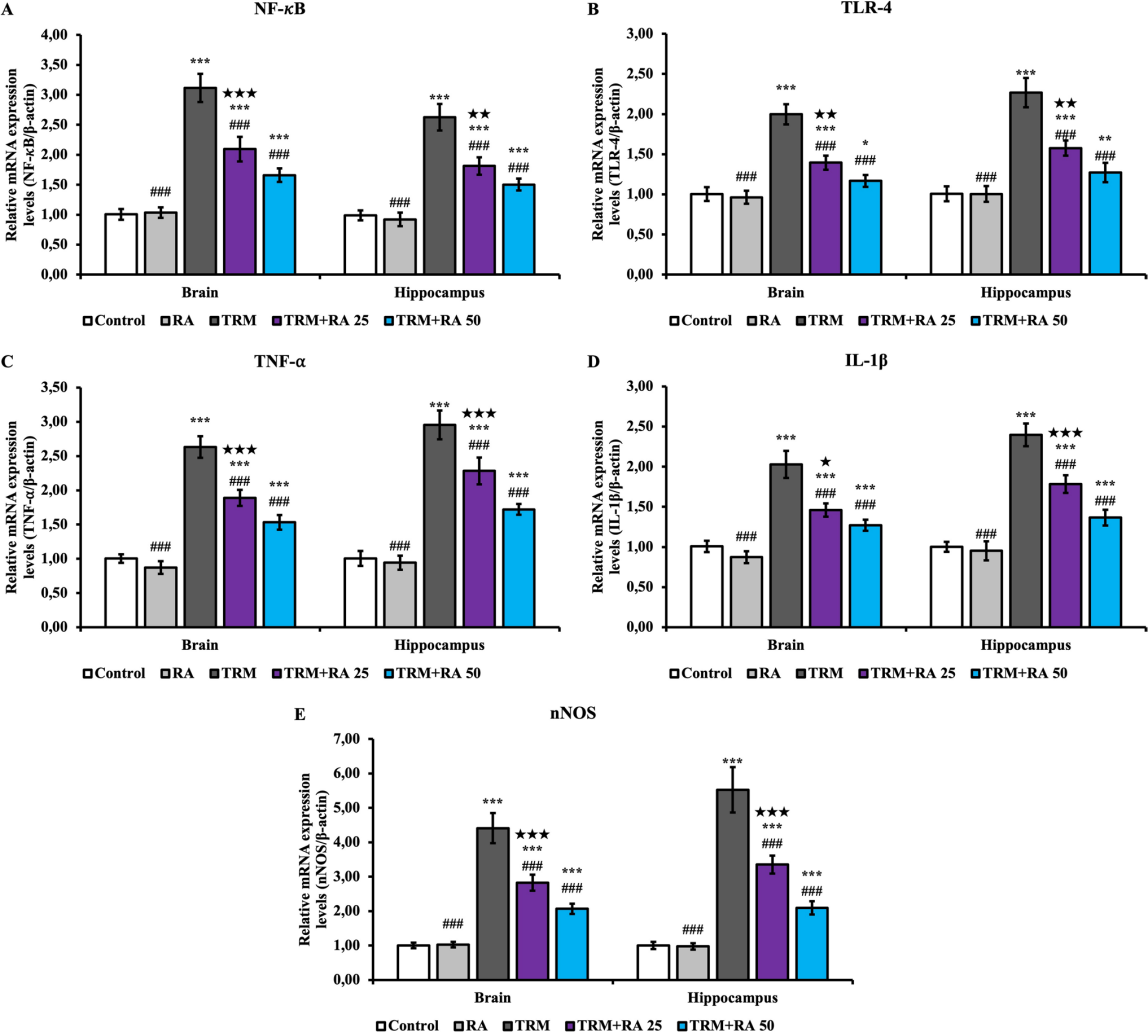
Effects of TRM and RA applications on NF-κB, TLR-4, TNF-⍺, IL-1β and nNOS mRNA transcription levels in brain and hippocampus tissues of rats. Statistical significance (Control vs others: *p < 0.05, **p < 0.01, ***p < 0.001, TRM vs others: #p < 0.05, ##p < 0.01, ###p < 0.001, TRM + RA 25 vs TRM + RA 50: ★p < 0.05, ★★p < 0.01, ★★★p < 0.001) was analyzed by One Way ANOVA

Nuclear factor kappa B (NF-κB) (Fig. 4A), toll-like receptor 4 (TLR-4) (Fig. 4B), tumor necrosis factor-⍺ (TNF-⍺) (Fig. 4C), IL-1β (Fig. 4D), and neuronal nitric oxide synthase (nNOS) (Fig. 4E) mRNA transcription levels were determined in brain and hippocampus tissues to determine the level of inflammation damage. TRM treatment increased the levels of NF-κB, TLR-4, TNF-⍺, IL-1β and nNOS in brain and hippocampus tissues compared with the control group (p < 0.001). Compared with the TRM group, the TRM + RA groups showed decreased levels of NF-κB, TLR-4, TNF-⍺, IL-1β and nNOS in brain and hippocampus tissues at both doses (p < 0.001). RA was more effective at higher doses (significance details are given in Fig. 4).

### RAGE Signaling Pathway Findings

**Fig. 5:**
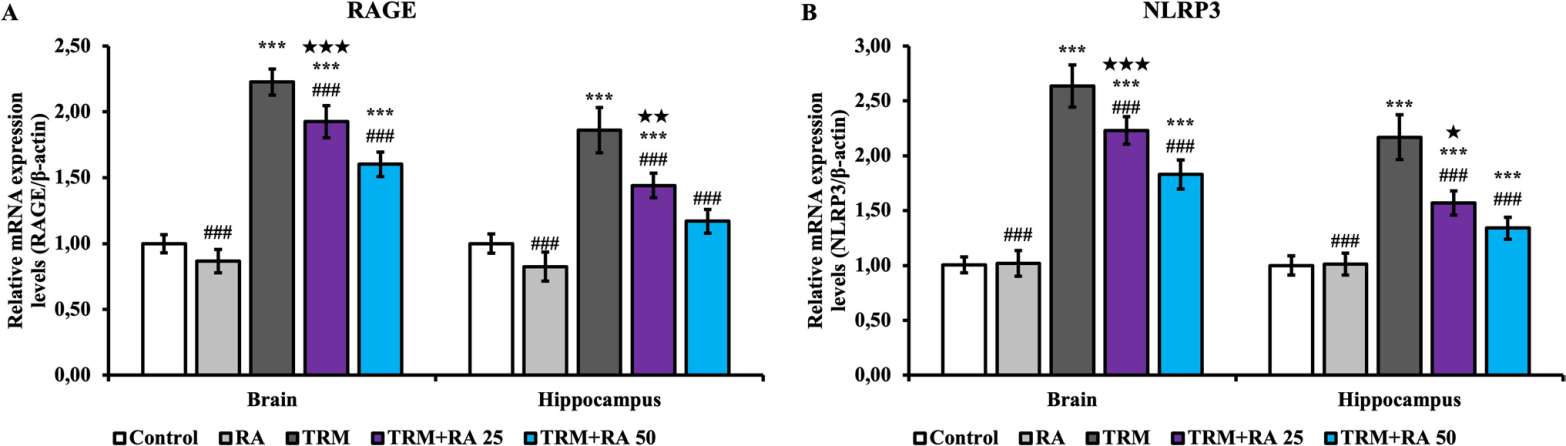
Effects of TRM and RA applications on RAGE and NLRP3 mRNA transcription levels in brain and hippocampus tissues of rats. Statistical significance (Control vs others: *p < 0.05, **p < 0.01, ***p < 0.001, TRM vs others: #p < 0.05, ##p < 0.01, ###p < 0.001, TRM + RA 25 vs TRM + RA 50: ★p < 0.05, ★★p < 0.01, ★★★p < 0.001) was analyzed by One Way ANOVA

Receptor for advanced glycation endproducts (RAGE) (Fig. 5A) and NLR family pyrin domain containing 3 (NLRP3) (Fig. 5B) mRNA transcription levels, which are other parameters that are effective in inflammation, were also determined. RAGE and NLRP3 mRNA transcription levels increased in brain and hippocampus tissues in the TRM group compared ​​​​​​​to the control group (p < 0.001). With RA administration, the effect of TRM was reversed, and RAGE and NLRP3 mRNA transcription levels decreased in both tissues (p < 0.001 at both doses). RA was more effective at a 50 mg/kg dose (significance details are given in Fig. 5).

### Apoptosis Findings

**Fig. 6:**
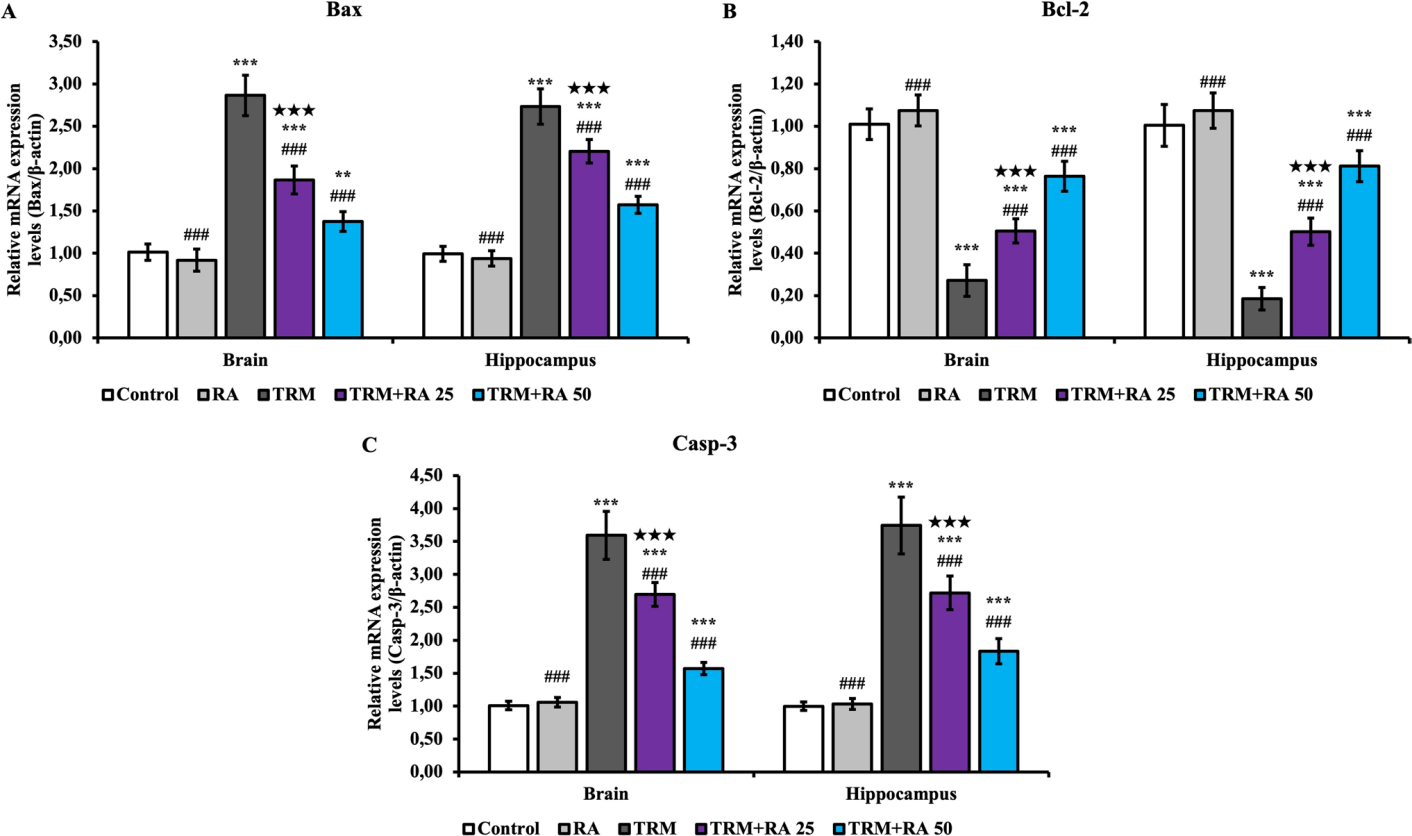
Effects of TRM and RA applications on Bax, Bcl-2, and Casp-3 mRNA transcription levels in brain and hippocampus tissues of rats. Statistical significance (Control vs others: *p < 0.05, **p < 0.01, ***p < 0.001, TRM vs others: #p < 0.05, ##p < 0.01, ###p < 0.001, TRM + RA 25 vs TRM + RA 50: ★p < 0.05, ★★p < 0.01, ★★★p < 0.001) was analyzed by One Way ANOVA

BCL2 Associated X (Bax) (Fig. 6A), B-cell lymphoma 2 (Bcl-2) (Fig. 6B), and cysteine-aspartic acid protease-3 (Casp-3) mRNA transcription levels were determined to determine the level of apoptotic damage in brain and hippocampus tissues. TRM treatment increased apoptotic Bax and Casp-3 levels in both tissues, whereas anti-apoptotic Bcl-2 levels decreased (p < 0.001). When RA was administered together with TRM, apoptotic Bax and Casp-3 levels decreased in both tissues, whereas anti-apoptotic Bcl-2 levels increased (p < 0.001). RA was more effective at a dose of 50 mg/kg (p < 0.001).

### BDNF mRNA Transcription Level Findings

**Fig. 7:**
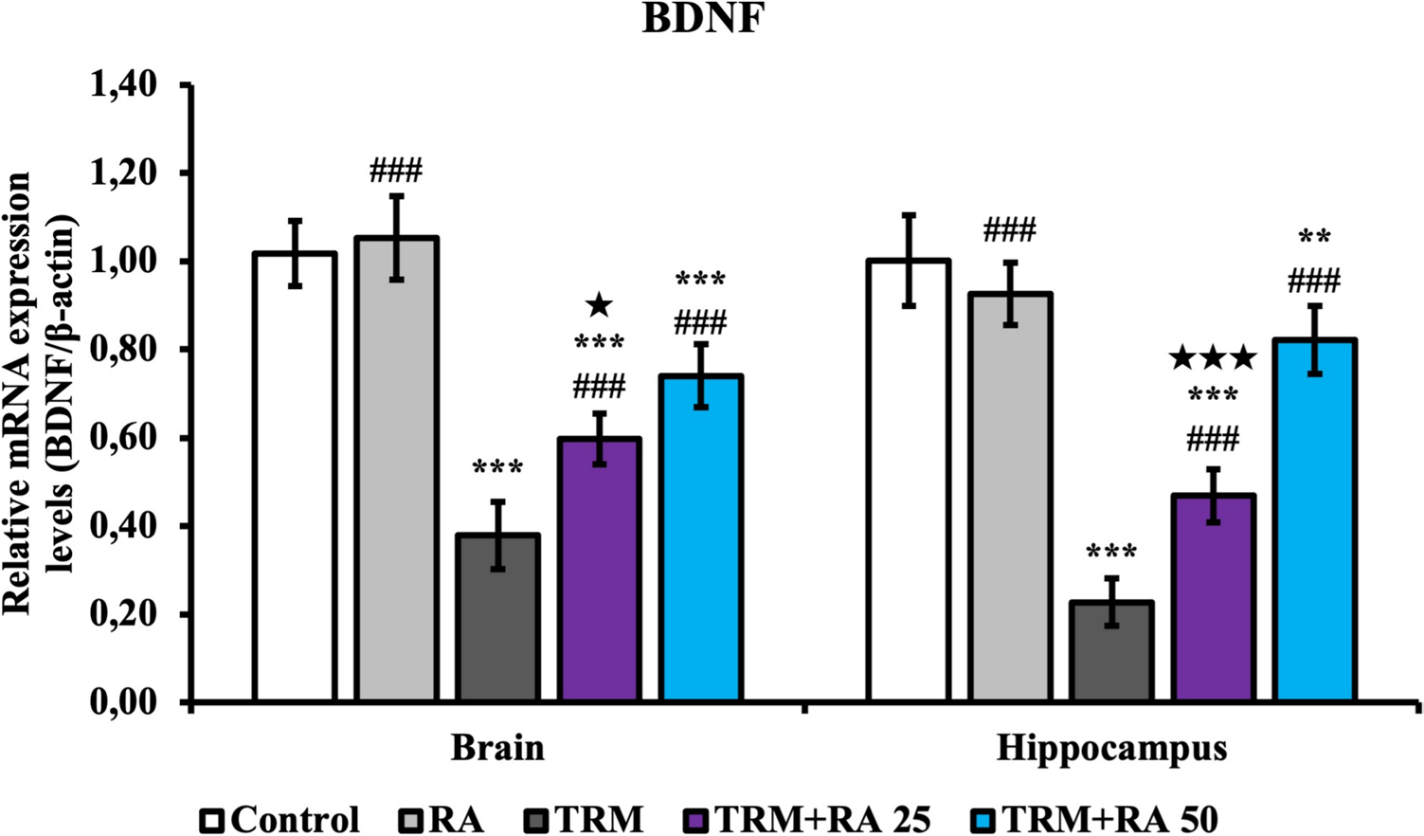
Effects of TRM and RA applications on BDNF mRNA transcription levels in brain and hippocampus tissues of rats. Statistical significance (Control vs others: *p < 0.05, **p < 0.01, ***p < 0.001, TRM vs others: #p < 0.05, ##p < 0.01, ###p < 0.001, TRM + RA 25 vs TRM + RA 50: ★p < 0.05, ★★p < 0.01, ★★★p < 0.001) was analyzed by One Way ANOVA

Brain-derived neurotrophic factor (Fig. 7) mRNA transcription levels, which play an important role in neuronal activities, were also determined. BDNF levels were lower in brain and hippocampus tissues in the TRM group than in the control group (p < 0.001). With RA treatment, BDNF levels increased in both tissues in the TRM + RA group compared with the TRM group (p < 0.001 at both doses). RA was more effective at a dose of 50 mg/kg (brain; p < 0.05, hippocampus; p < 0.001).

### Light Microscopy Examinations

**Fig. 8:**
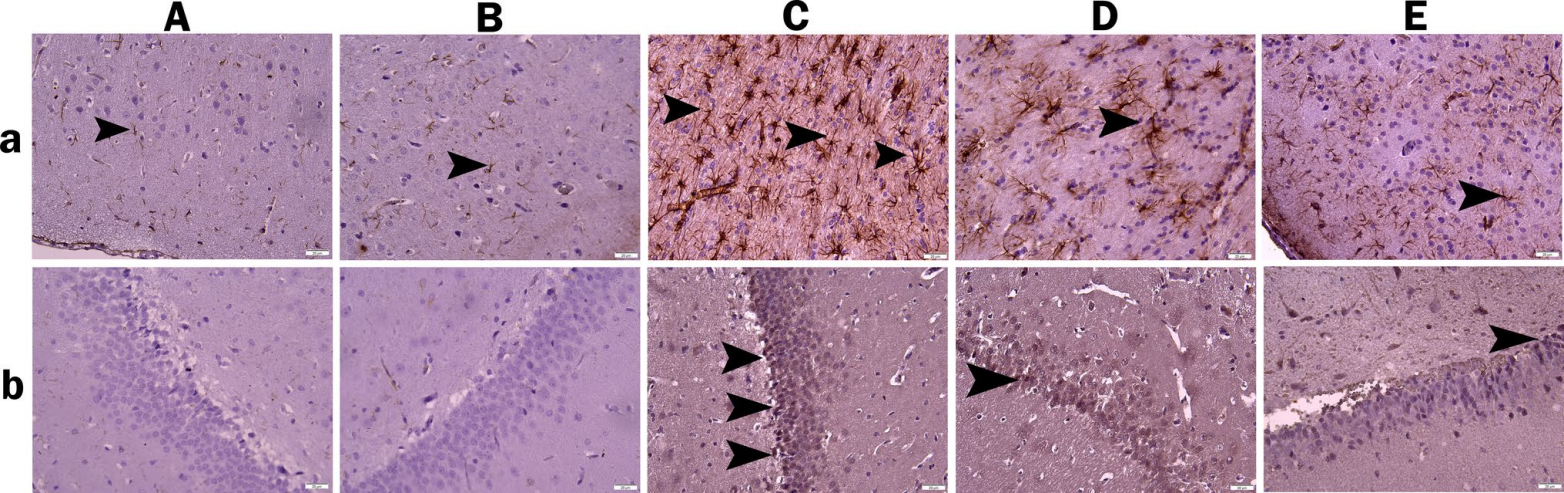
Photomicrograph of cerebral cortex (a) and hippocampus (b) of hematoxylin and eosin stained brain sections (Bar: 50 μm). Control (A) and RA (B) groups show healthy histological arrangement and architecture of the cerebral cortex. TRD group (C) shows vacuolation in the cytoplasm of the cells, pyknotic changes and degeneration in neurons, and loss of hippocampus neurons in addition to vascular congestion. The TRD + RA 25 group (D) and the TRD + RA 50 group; although close to the normal histological arrangement, show vacuolation in the cytoplasm of some cells, vascular congestion in the vessels, and mild atrophy and degeneration in neurons. arrowhead: vacuolation, star: vascular congestion, arrow: pyknotic changes and degeneration in neurons, curved arrow: neuronal cell loss

When the histological results of brain tissue from the control and RA groups were analyzed, the cerebral cortex showed normal cellular structure, with no pathological findings. When the hippocampus was examined, the general morphology of neurons and glial cells was normal (Fig. 8A, Fig. 8B). In the TRM group, hyperemia and congestion were observed in the vessels of the meningeal and cerebral cortex. In addition, pyknotic changes, an increase in hyperchromatic nuclei, and vacuolization in the cytoplasm of some cells were detected in neurons. Therefore, atrophy and degeneration were observed in neurons. In the hippocampus of the TRM group, cell organization was disrupted, and the layer thickness was decreased. Vacuolization in cells and intense pyknotic changes in nuclei were observed (Fig. 8C). In the TRM + RA 25 group, neurons in brain tissue exhibited pyknotic changes, degeneration in a few cells, and mild hyperemia in the vessels. The cells in the hippocampus of this group showed moderate degenerative changes and dark pyknotic nuclei in some cells (Fig. 8D). In the TRM + RA 50 group, the normal histological structure of the cerebral cortex layer was regained, except for mild hyperemia and cellular degeneration in rare areas. The hippocampus had a near-normal appearance, except for mild pyknotic changes (Fig. 8E). The results are summarized in Table 2.

**Table 2:** Histopathological and immunohistochemical findings and scores in rat cerebral cortex and hippocampus tissue

Parameters	Control	RA	TRM	TRM + RA 25	TRM + RA 50
**Pycnotic changes and degeneration in cells**	–	–	+ + +	+	+
**Vascular hyperemia and congestion**	–	–	+ + +	+	+
**GFAP expression**	+	+	+ + +	+ +	+
**Caspase 3 expression**	–	–	+ + +	+	+

( −) No change, ( +) Slight change, (+ +) Moderate change, (+ + +) Severe change

### Immunohistochemical Results

**Fig. 9:**
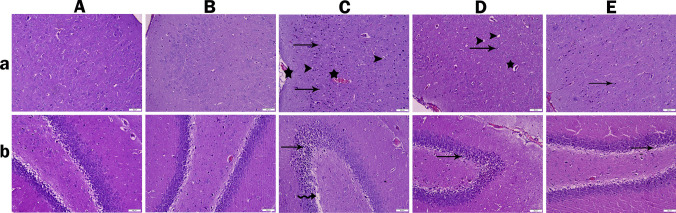
Photomicrograph of brain sections stained with immunohistochemical technique for GFAP in the cerebral cortex (a) and Casp-3 in the hippocampus (b) (Bar: 20 μm). Control (A) and RA (B) groups show low expression of GFAP in the cerebral cortex. The TRD group (C) shows intense positive immunoreactivity for GFAP in the cerebral cortex and Casp-3 in the hippocampus. TRD + RA 25 group (D) shows moderate positive immunoreaction for GFAP in the cerebral cortex and mild positive immunoreaction for Casp-3 in the hippocampus. TRD + RA 50 (E) group shows slightly positive immunoreaction of GFAP in the cerebral cortex and Casp-3 in the hippocampus. Arrowhead: shows positive immunoreactive expression of GFAP and Casp-3

When the glial fibrillary acidic protein (GFAP) immunohistochemistry results in brain tissue were analyzed, very mild positivity was found in the control and RA groups.​​​​​​​ When Casp-3 expression in the hippocampus was analyzed, no positivity was found (Fig. 9A, 9B). In the TRM-treated group, intense positivity was observed in brain tissue due to the overexpression of GFAP. Casp-3 expression was also found to be severe in the hippocampus of this group (Fig. 9C). In the TRM + RA 25 group, moderate positivity was observed due to a decrease in brain GFAP expression compared with the TRM-only group. Similarly, mild positivity was observed in the hippocampus due to decreased Casp-3 expression (Fig. 9D). In the TRM + RA 50 group, there was a greater dose-dependent decrease in GFAP positivity. Casp-3 expression in the hippocampus of this group was mild (Fig. 9E). The results are summarized in Table 2.

#### Western Blot Results

**Fig. 10:**
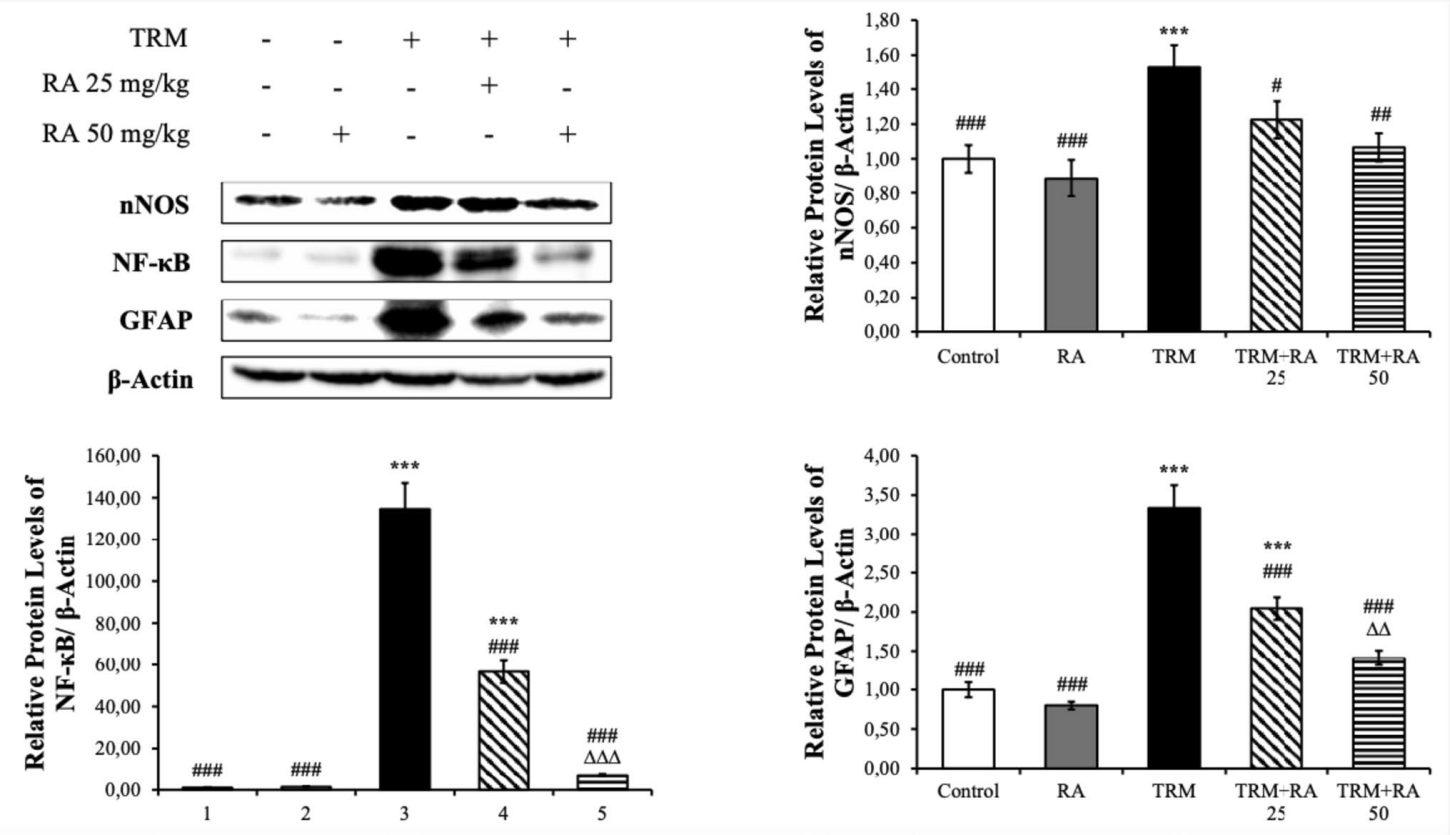
Effects of TRM and RA administrations on nNOS, NF-κB and GFAP protein levels in brain tissues of rats. Values are given as mean ± SD. Control vs others: *p < 0.05, **p < 0.01, ***p < 0.001, TRM vs others: #p < 0.05, ##p < 0.01, ###p < 0.001, TRM + RA 25 vs TRM + RA 50: ∆p < 0.05, ∆∆p < 0.01, ∆∆∆p < 0.001

**Fig. 11:**
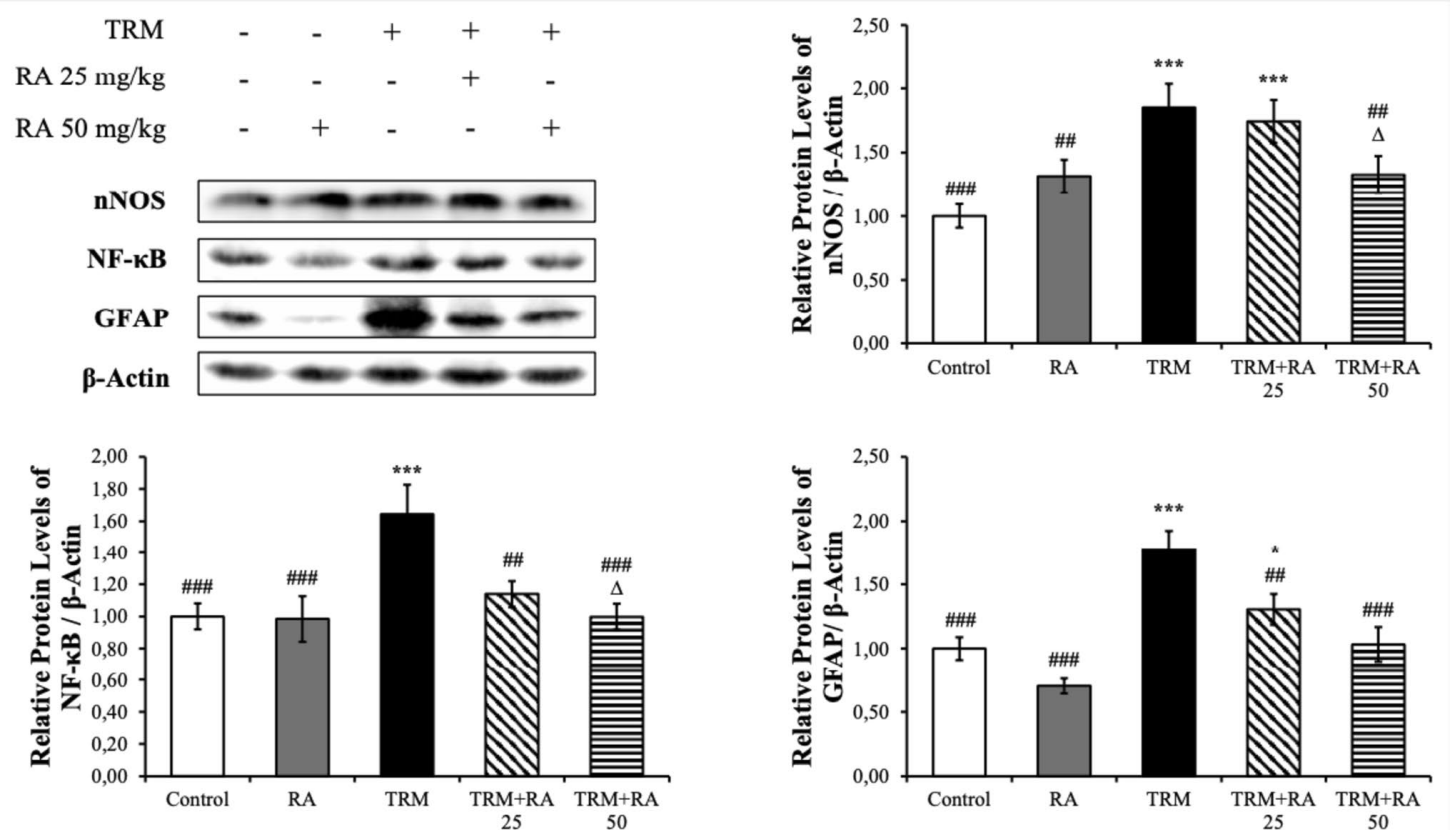
Effects of TRM and RA administrations on nNOS, NF-κB and GFAP protein levels in hippocampus tissues of rats. Values are given as mean ± SD. Control vs others: *p < 0.05, **p < 0.01, ***p < 0.001, TRM vs others: #p < 0.05, ##p < 0.01, ###p < 0.001, TRM + RA 25 vs TRM + RA 50: ∆p < 0.05, ∆∆p < 0.01, ∆∆∆p < 0.001

The nNOS, NF-κB and GFAP protein levels in brain (Fig. 10) and hippocampus (Fig. 11) tissues were also analyzed.

TRM treatment increased nNOS, NF-κB and GFAP protein levels in brain tissue samples (p < 0.001). With RA treatment, TRM-induced increases were stopped and decreased by moving in the opposite direction (nNOS; RA 25 mg/kg dose: p < 0.05, RA 50 mg/kg dose: p < 0.01. Others; p < 0.001). TRM increased nNOS, NF-κB and GFAP protein levels in hippocampus tissues (p < 0.001). When RA was administered together with TRM, nNOS, NF-κB and GFAP protein levels were decreased compared with the TRM group. This significance was p < 0.01 for NF-κB and GFAP at a dose of 25 mg/kg dose, p < 0.01 for nNOS and p < 0.001 for NF-κB and GFAP at 50 mg/kg. RA was more effective in the decrease in nNOS and NF-κB at 50 mg/kg dose (p < 0.05).

## Discussion

This study investigated the neurotoxic effects of TRM that may occur due to abuse and overdose. Another aim was to investigate the role of RA in alleviating tissue damage caused by RA, despite the toxic effects of TRM. For these purposes, the analysis of important parameters that play a role in oxidative stress, inflammation, ER stress, apoptosis in brain and hippocampus tissues, cognitive function analysis, and structural and functional changes in tissues was performed.

TRM impairs cognitive function in the cerebral cortex in both abuse and overdose. Furthermore, this negative effect is at its peak when combined with other abused substances [25, 26]. In this study, TRM application negatively affected the cognitive function of rats, as assessed using the MWT. Although the results of the neurobehavioral assessment did not reach statistically significant levels, a trend towards impairment was observed. For example, the first reaching time to the platform quadrant during the probe trial was longer compared with that of the healthy group, and the healthy group spent more time in the platform quadrant. Another finding was that the time to reach the platform slightly decreased during the acquisition phase in the TRM group but gradually decreased in healthy rats. Similar to the current study, Ishola et al. [27] reported that TRM use negatively affected cognitive function. On the other hand, the RA application appeared to counteract the effects of TRM, showing a trend toward improving cognitive function in all measured parameters. This lack of statistical significance might be explained by the limited duration of RA administration or the possibility that a longer recovery period is needed for cognitive improvement to reach measurable levels. Another supportive result is that there was no difference in average velocity between the groups in the 4th-day test, indicating that the groups were similar in terms of motor skills or general physical capacity. This suggests that the differences in cognitive performance between the groups were not due to motor or physical capacity, but mainly due to cognitive processes (learning and memory).

The brain is highly susceptible to oxidative damage due to limited antioxidant defense mechanisms despite high oxygen consumption [9]. Oxidative stress occurs as a result of disruption of the balance between antioxidants and increased ROS [28]. Increased levels of ROS can damage proteins in many tissues, and they are the primary cause of tissue damage [29, 30]. With increased oxidative stress, many tissue damage signaling pathways, such as inflammation and apoptosis, can be activated; making it possible for many diseases, such as neurodegenerative disorders, to occur [31]. Lipids forming the cell membrane are directly affected by the increase in ROS and are subjected to lipid peroxidation, resulting in the formation of products such as MDA resulting from the metabolism of polyunsaturated fatty acids. MDA is an important biochemical indicator of oxidative stress [32]. Antioxidant enzymes are critical components of the defense against oxidative stress. [33, 34]. SOD converts superoxide to hydrogen peroxide (H₂O₂), while CAT decomposes H₂O₂ into water and oxygen. GPx neutralizes both lipid peroxides and H₂O₂. [35]. GSH also binds free radicals and converts them into oxidized form, thereby supporting antioxidant defense [36]. In this study, TRM treatment increased MDA levels, an important marker of lipid peroxidation, in brain and hippocampus tissues. At the same time, the activities of antioxidant defense enzymes such as SOD, CAT and GPx as well as reduced GSH levels were significantly decreased. These changes triggered oxidative stress by disrupting the oxidant-antioxidant balance in favor of oxidants in TRM, brain and hippocampus tissues. However, when RA was co-administered with TRM, it showed a potent antioxidative effect. RA significantly reduced MDA levels, indicating a decrease in lipid peroxidation and oxidative damage. Furthermore, RA potentiated the antioxidant defense system by increasing SOD, CAT and GPx activities and GSH levels. These findings suggest that RA not only reduces TRM-induced oxidative damage but also supports antioxidant capacity, thereby providing neuroprotection against oxidative stress in brain and hippocampus tissues. Similar to this study, Ma et al. [37] reported that RA exhibited healing properties by reducing oxidative stress in the spinal cord in their study of spinal cord paralyzed rats.

Increased oxidative stress can trigger inflammatory responses and ER stress, which are associated with mitochondrial dysfunction [38]. Normally, protein synthesis in the ER is managed in harmony with cellular needs, ensuring proper folding of proteins. However, under adverse conditions, the protein folding load increases, leading to the accumulation of misfolded proteins and ER stress [39]. The key determinants of ER stress in mammalian cells are IRE1, PERK and ATF-6 [40, 41]. Increases in the number of misfolded proteins activate ER-mediated apoptosis is activated [42]. ATF-4 is a transcription factor that activates genes that control cellular activities such as protein folding, amino acid metabolism, and apoptosis [43]. With the activation of PERK, eIF2-α is phosphorylated, which suppresses protein synthesis and folding [44]. It has been reported in different studies that various toxic substances can cause ER stress [40]. In this study, TRM-induced ER stress by increasing eIF2-α, ATF-4, PERK, ATF-6, and IRE1 mRNA levels in brain and hippocampus tissues. In contrast, treatment with RA significantly alleviated TRM-induced ER stress by reducing the expression levels of eIF2-α, ATF-4, PERK, ATF-6, and IRE1 mRNA in both brain and hippocampus tissues. These findings suggest that RA exerts a protective role against ER stress, likely by mitigating protein misfolding and restoring cellular homeostasis.

If oxidative stress continues, it will eventually lead to increased inflammatory damage [45]. When NF-κB is activated, it dissociates from IκB and moves from the cytoplasm to the nucleus, where it controls the expression of approximately 500 genes. These genes include those responsible for the production of proinflammatory cytokines such as TNF-α and IL-1β, which drive the inflammatory process [46–48]. IL-1β, produced by macrophages, is an important cytokine that mediates inflammation [47]. The effects of nitric oxide (NO) depend on the enzyme that triggers its production, and the consequences range from protective to harmful [49]. The three isoforms of nitric oxide synthase (NOS), nNOS, endothelial NOS (eNOS), and inducible NOS (iNOS), are closely associated with the development and maintenance of neuropathic pain. In particular, nNOS overexpression plays an important role in pathological processes such as neurotoxicity, septic shock, and neuropathic pain, and it plays a critical role in central sensitization mechanisms [50]. In addition, there is increasing evidence that TLR4 plays a pronociceptin role together with RAGE [51]. In the current study, the levels of NF-κB, TLR-4, TNF-α, IL-1β, and nNOS, which play an active role in inflammation and pathological processes, increased with TRM administration, and inflammatory damage occurred in the brain and hippocampus tissues. At the same time, an increase in pathological neuronal processes was observed. RA treatment reversed this effect of TRM and reduced NF-κB, TLR-4, TNF-α, IL-1β and nNOS levels, alleviating inflammatory damage. A similar situation was observed at the NF-κB protein level. Furthermore, RA treatment not only reduced the levels of these inflammatory markers but also appeared to mitigate the pathological neuronal processes observed in brain and hippocampus tissues following TRM administration. By modulating the NF-κB pathway and significantly downregulating TLR-4, TNF-α, IL-1β, and nNOS expression, RA effectively disrupted the cascade of inflammatory responses. This dual action—reducing inflammation and alleviating pathological neuronal changes—highlights the therapeutic potential of RA. These findings suggest that RA not only alleviates inflammation but may also play a crucial role in protecting neuronal structures and restoring tissue homeostasis in TRM-induced conditions. Similar to our study, Thingero et al. [52] reported that RA exhibits protective properties against neuroinflammation by inhibiting proinflammatory cytokines.

RAGE, which plays an important role in accelerating the inflammatory process, activates the NF-κB signaling pathway, whereas the inflammasome, which consists of protein complexes such as NLRP3 that regulate inflammation, is activated by exogenous stimuli and promotes the maturation and release of proinflammatory cytokines [53] NLRP3 is an important component of the innate immune system that responds to stress factors and intracellular infections. NLRP3 is a multiprotein immune sensor complex comprising the adaptor subunit (ASC) and theprospase-1 subunit. Although it has been shown that advanced glycation end products (AGEs) can induce NLRP3 inflammasome activation during inflammation, the mechanism underlying this activation is related to the perception of cellular stress signals, particularly ROS overproduction and RAGE overexpression. However, this mechanism has not yet been fully elucidated [38]. In this study, RAGE and NLRP3 mRNA transcription levels increased in brain and hippocampus tissues following TRM. RA treatment reversed the effect of TRM and exerted protective effects by decreasing RAGE and NLRP3 levels in brain and hippocampus tissues. Furthermore, RA treatment not only reduced the transcription levels of RAGE and NLRP3 but also likely modulated upstream signaling pathways, such as ROS production, to attenuate cellular stress responses. By downregulating the key mediators of the inflammatory cascade, RA effectively disrupted the feedback loop between oxidative stress and inflammasome activation. These protective effects highlight RA's potential in mitigating inflammation-driven damage and maintaining cellular homeostasis in brain and hippocampus tissues under TRM-induced pathological conditions. This suggests that RA may serve as a valuable therapeutic agent in preventing the progression of RAGE- and NLRP3-mediated inflammatory processes.

In the absence of external stimuli, caspase activity is typically suppressed. When apoptosis begins, caspases activate each other sequentially, forming a cascade [54]. In healthy cells, apoptosis is usually triggered by risk factors such as cellular stress or injury [55]. The production of ROS occurs mostly in mitochondria, and increased ROS levels are an important component of the apoptotic cascade [56]. In addition, it has been proven that TNF-α, a pro-inflammatory molecule, induces apoptosis [57]. Two important proteins in cells participate in competitive interactions on the mitochondrial membrane: proapoptotic Bax and antiapoptotic Bcl-2. The apoptotic process is shaped by the balance between these two proteins; the Bax/Bcl-2 ratio is considered an important indicator of apoptosis. An imbalance in this ratio accelerates apoptosis and programed death of cells. On the other hand, an imbalance in favor of Bcl-2 ensures cell survival [58]. In this study, we revealed that TRM-induced neurotoxicity increased the mRNA transcription levels of apoptotic factors Casp-3 and Bax in brain and hippocampus tissues and decreased the mRNA transcription level of Bcl-2, which has an antiapoptotic effect. On the other hand, it was observed that RA reversed these negative effects caused by TRM, decreased the mRNA expression of Casp-3 and Bax, and increased the mRNA transcription level of Bcl-2. In addition, a similar situation was detected via immunohistochemical analysis. These findings suggest that RA exerts its neuroprotective effects by modulating the balance between proapoptotic and antiapoptotic factors, thereby mitigating TRM-induced apoptotic damage in brain and hippocampus tissues. The decrease in Casp-3 and Bax expression, along with the increase in Bcl-2 levels, indicates that RA not only suppresses apoptosis but also promotes cell survival under conditions of oxidative stress and neurotoxicity. Furthermore, the consistency between molecular findings and immunohistochemical results reinforces the therapeutic potential of RA in preserving neuronal integrity. By restoring the Bax/Bcl-2 ratio and inhibiting the activation of apoptotic pathways, RA demonstrates its ability to counteract TRM-induced neurotoxicity, highlighting its promise as a protective agent against apoptosis-driven neurodegeneration. Similar to our study, Khamse et al. [59] reported that RA protects neurons against apoptosis by inhibiting the apoptotic cascade and increasing Bcl activity.

BDNF is an important neurotrophic factor that participates in various intracellular signaling processes, neuronal protection and survival, dendritic and axonal morphology, and synaptic plasticity [60]. BDNF is the most abundant neurotrophin in the central nervous system and plays an important role in neuronal activity by supporting neuronal survival; it is associated with learning, memory, and neurodegenerative diseases [60]. In this study, TRM decreased BDNF levels in brain and hippocampus tissues, negatively affected neuronal survival and activity, and caused neurotoxicity. RA reversed these TRM-induced effects, increased BDNF levels. These results highlight the critical role of RA in preserving neuronal function and promoting recovery in TRM-induced neurotoxicity. By increasing BDNF levels, RA not only counteracts the detrimental effects of TRM on neuronal survival and activity but also enhances synaptic plasticity and supports overall neuronal health. The restoration of BDNF levels suggests that RA may facilitate neuroprotection and cognitive resilience by fostering the growth and maintenance of neuronal networks. These findings underscore RA’s therapeutic potential in mitigating neurotoxicity and improving brain function, particularly in conditions where neurotrophic support is compromised.

TRM can accelerate neurodegeneration in many areas of the brain, leading to various behavioral disorders and histopathological changes. Rapidly absorbed and rapidly crossing the blood–brain barrier when taken orally, TRM has the potential for various effects on the central nervous system [61]. Studies examining its effects on the brain have reported that TRM exhibits serious neurotoxic effects, including neuronal degeneration, inflammation, gliosis, and hippocampal damage [62]. In this study, TRM-induced histological damage occurred in both brain and hippocampus tissues. In particular, neuronal atrophy and degeneration, disruption of cell organization, and pyknotic changes were observed. RA exhibited protective properties by attenuating all TRM-induced structural changes. These findings emphasize RA’s significant neuroprotective role in preventing structural damage caused by TRM. By reducing neuronal atrophy and degeneration, preserving cell organization, and minimizing pyknotic changes, RA demonstrated its ability to safeguard the integrity of both brain and hippocampus tissues. This protective effect suggests that RA not only counteracts the immediate histopathological damage induced by TRM but may also help maintain the functional architecture of neural networks. Consequently, RA holds promise as a therapeutic agent capable of mitigating neurodegenerative processes and preserving brain health in conditions associated with TRM-induced toxicity. Similar to this study, Hassanzadeh-Taheri et al. [63] reported that RA increased the number of neurons and decreased the number of damaged neurons in hippocampus regions. Another similarity with this study is that these effects of RA were more pronounced at doses of 50 mg/kg.

GFAP is an intermediate filament protein found primarily in astrocytes, and its elevated serum levels are used as a biomarker for the detection of cerebral hemorrhage in the acute phase of stroke, traumatic brain injury, and traumatic spinal cord injury [64]. Because GFAP expression increases in response to brain injury, it is considered a biomarker of CNS damage [65]. In this study, GFAP protein levels increased in brain and hippocampus tissues following TRM administration. These findings may prove that TRM causes significant damage to brain and hippocampus tissues. Conversely, RA prevented TRM-induced damage to brain and hippocampus tissues by decreasing GFAP protein levels. A similar situation was detected in the immunohistochemical analysis. These results highlight RA's potential to mitigate the neuroinflammatory response triggered by TRM-induced brain injury. By decreasing GFAP protein levels, RA effectively reduces astrocyte activation, which is often a hallmark of neuroinflammation and central nervous system damage. This suggests that RA not only alleviates the inflammatory response but also helps restore normal tissue architecture in brain and hippocampus regions affected by TRM. The consistency between the molecular data and immunohistochemical findings further supports RA's protective properties, positioning it as a promising therapeutic agent to counteract the damage caused by neurotoxic substances like TRM.

## Conclusion

As a result, TRM may cause deterioration in learning and memory by triggering oxidative stress, inflammation, endoplasmic reticulum stress, and apoptosis in the brain and hippocampus tissues of rats and by disrupting tissue structural and functional properties. RA, particularly at its more effective dose of 50 mg/kg, can be considered an effective alternative for preventing and reducing TRM-induced neurotoxicity because of its protective effect against such damage.

## Data Availability

No datasets were generated or analysed during the current study.

## Declarations

### Clinical Trial

Clinical trial number: not applicable.
